# Retirement of Mary Traynor, executive editor of JXB (1995–2020)

**DOI:** 10.1093/jxb/eraa314

**Published:** 2020-09-09

**Authors:** Bill Davies, Jerry Roberts, Christine Raines, John E Lunn

**Affiliations:** 1 Lancaster Environment Centre, Lancaster University, Lancaster, United Kingdom; 2 University of Plymouth, Drake Circus, Plymouth, Devon, United Kingdom; 3 University of Essex, Wivenhoe Park, Colchester, United Kingdom; 4 Max Planck Institute of Molecular Plant Physiology, Potsdam-Golm, Germany

**In July 2020 we bid a fond farewell to Mary Traynor, who is retiring as Executive Editor of the *Journal of Experimental Botany*, after 25 years’ service as head of the journal’s office in Lancaster. Mary joined the journal in 1995 while completing her PhD (‘Root growth in drying soil: a role for ABA?) under the supervision of Bill Davies. Bill was Editor-in-Chief of *JXB* from 1995 to 2007 and appointed Mary to the post of Executive Editor (then called Assistant Editor), when the previous incumbent, Sarah Blackford, decided to follow a different career path. Bill recognised that Mary’s enthusiasm for plant science and understanding of its value to society for addressing global challenges would be tremendous assets for both *JXB* and its parental organisation, the Society for Experimental Biology (SEB).**

The intervening years have seen enormous changes and challenges in the world of scientific publishing with the digital revolution and increased competition from new journals. Yet under Mary’s wise and inspiring leadership, the journal has not only survived but thrived, growing in scientific reputation and reaching an ever-wider international audience, while at the same time providing valuable income for the SEB to support its activities in the scientific community. In the early years, Mary and Bill launched the journal’s Special Issues, which were initially linked to symposia at SEB meetings. These and other new features, such as the Flowering Newsletter, that were instituted during Mary’s time in office have made a major contribution to the success of the journal. In partnership with Bill’s successors as Editor-in-Chief, Jerry Roberts (2008–2012) and Christine Raines (2012–2020), Mary played a leading role in other important innovations, including the early adoption of Open Access for authors from subscribing institutions, the development of the eXtra Botany section and Darwin Reviews, and the creation of a strong online presence. Mary and her team also helped *JXB* to become a truly global plant science journal by encouraging the appointment of overseas editors and promoting the journal at international conferences.

These achievements reflect Mary’s strong scientific background, sound judgement, organisational skills, understanding of the publishing world and, above all, commitment to *JXB* and its ethos as a community-based journal. Underpinning these professional attributes and a key to her success are Mary’s personal qualities, which are reflected in the warm and affectionate comments we received from many of her past and present colleagues from the journal office. She is above all a kind and generous person who genuinely cares about people, social issues and nature. Over the years she has displayed a remarkable talent for spotting potential in people, nurturing and inspiring younger members of staff with her kindness, good humour and wisdom. By giving each person a voice and demonstrating her trust in their abilities, Mary created a happy and supportive environment in which her junior colleagues flourished, with several of her protégés advancing to senior editorial positions with other journals. Colleagues from the SEB and our publisher, Oxford University Press, also expressed their warm appreciation for Mary’s abilities and character, recognising her genuine desire and determination to make things better for the journal. Never shy to protect the journal’s interests, she has always done so with professionalism, a positive attitude and good humour. The journal’s authors and scientific editors have also benefitted greatly from Mary’s sage advice over the years, based on her ability to see both sides of an argument, make sound judgements, and express her views in a tactful and constructive way.

As past and current Editors-in-Chief of *JXB*, we owe a particular debt of gratitude to Mary for her invaluable support in helping us to meet the many challenges of the job, and it is clear to all of us how much the success of *JXB* over the last 25 years has depended on Mary as the lynchpin between the scientific, administrative and publishing arms of the journal. On behalf of all our authors, editors, readers and staff, we wish to express our heartfelt thanks to Mary for her superb and devoted service to the journal, and we wish her a long and happy retirement to enjoy watching her grandchildren grow up and share her passion for the natural world and the hills of the Lake District.


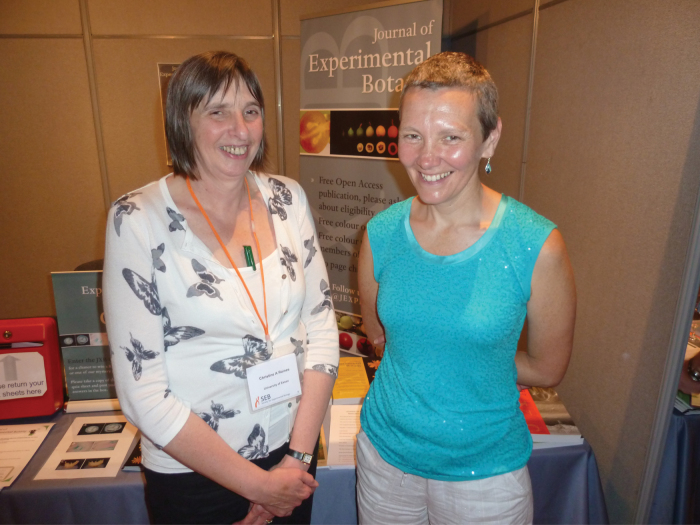


Mary (right), with Christine Raines, at the SEB Meeting in Prague, July 2015.

